# Use of mobile phones to collect data on COVID-19: phone access and participation rates, in Rakai, Uganda

**DOI:** 10.1080/16549716.2024.2419160

**Published:** 2024-11-12

**Authors:** Robert Ssekubugu, Anthony Ndyanabo, Fredrick Makumbi, Anna Mia Ekström, Laura Beres, Grace Nalwoga Kigozi, Hadijja Nakawooya, Joseph Ssekasanvu, Maria J Wawer, Fred Nalugoda, Nelson Sewankambo, Victor Ssempijja, Betty Nantume, David Serwadda, Godfrey Kigozi, Ronald H. Gray, Larry W. Chang, M. Kate Grabowski, Helena Nordenstedt, Joseph Kagaayi

**Affiliations:** a Rakai Health Sciences Program, Kalisizo, Uganda; b Department of Global Public Health, Karolinska Institutet, Stockholm, Sweden; c School of Public Health, College of Health Sciences, Makerere University, Kampala, Uganda; d Department of Infectious Diseases/Venhälsan, South General Hospital, Stockholm, Sweden; e Department of International Health, Johns Hopkins Bloomberg School of Public Health, Baltimore, MD, USA; f Department of Epidemiology, Johns Hopkins Bloomberg School of Public Health, Baltimore, MD, USA; g School of Medicine, College of Health Sciences, Makerere University, Kampala, Uganda; h Clinical Monitoring Research Program Directorate, Frederick National Laboratory for Cancer Research, Frederick, MD, USA; i Department of Pathology, Johns Hopkins School of Medicine, Baltimore, MD, USA; j Division of Infectious Diseases, Department of Medicine, Johns Hopkins School of Medicine, Baltimore, Maryland, MD, USA; k Department of Medical Specialties, Danderyd University Hospital, Stockholm, Sweden

**Keywords:** Mobile phone surveys, face-to-face interviews, sampling variation, participation rate, COVID-19, Rakai

## Abstract

During the COVID-19 pandemic lockdown, we deployed a rapid, mobile phone-based survey to assess access and participation rates when using mobile phones to collect data on COVID-19 in Rakai, south-central Uganda. We sampled prior Rakai Community Cohort Study (RCCS) participants based on HIV status using mobile phone contacts. We administered a 30-minute phone-based interview to consenting participants to assess their knowledge about different aspects of COVID-19 and their access to care. Our analysis compares the mobile phone survey participation rates with historic participation rates in regular RCCS face-to-face interviews. We supplemented phone survey data with demographic, behavioral, and HIV status data from prior face-to-face RCCS surveys. Phone access in Round 19 of the RCCS was found to be 90.2%, with lower access among older people, and people living with HIV. When including only individuals who participated in the previous RCCS survey round, participation in the face-to-face survey (81.9%) was higher than participation in our phone survey (74.8%, *p* < .001). Survey participation was higher among people living with HIV compared to HIV-negative individuals (84.0% vs 81.4%, *p* < .001) in the face-to-face survey, but in the phone survey the reverse was found, with participation rates being higher among HIV-negative individuals compared to people living with HIV (78.0% vs 71.6%, *p* < .001). It was possible to collect data from an existing population cohort during the lockdown using phones. Phone access was high. Overall participation rates were somewhat lower in the phone survey, notably in people living with HIV, compared to the face-to-face survey.

## Paper Context

- **Main findings**: It was feasible to conduct a phone survey within an existing population-based cohort in rural Uganda during the COVID-19 lockdown.
- **Added knowledge**: During a public health emergency, due to high levels of phone access within the population in rural Uganda, using a mobile phone survey to rapidly collect demographic and health data is possible, but it might yield somewhat lower participation rates, especially in fishing communities and among people living with HIV.
- **Global health impact for policy and action**: Collecting data face-to-face in population-based cohorts is cumbersome and costly. Switching partly to mobile phone surveys might be a way forward to maintain the frequency and intensity of survey rounds. Continuous monitoring of who is missing in the phone survey is critical to minimize biased interpretation of results.

## Background

Lockdowns during the COVID-19 pandemic presented a challenge to population surveillance efforts which use face-to-face interviews to assess population-level effects and tailor interventions. Uganda had one of Africa’s strictest responses to the COVID-19 pandemic [1]. Government-enforced restrictions on transportation and mobility, and prolonged school lockdowns impacted healthcare service delivery [2–5]. Uganda reported its first confirmed case of SARS-CoV-2 on 21 March 2020, after the World Health Organization designated COVID-19 a global pandemic [6,7]. Uganda’s first wave of COVID-19 was from March 2020 to January 2021 and the second wave peaked in June 2021 [8]. COVID-19 lockdowns precluded face-to-face community interviews, leaving mobile phone surveys as the primary mode of population surveillance [9,10].

Mobile phones are potential tools for rapidly gathering data during public health emergencies, [11–13] but it is unclear whether phone survey results are generalizable to the broader population [14]. To inform future research methodology, it is important to understand how the demographics and characteristics of mobile phone survey respondents compare with those of face-to-face survey respondents. This could be useful in ongoing disease surveillance in Africa [15–20].

Mobile phone surveys provide an opportunity to characterize population- and individual-level behaviors, but there is uncertainty about how to appropriately account for potential biases within these data. This study aims to identify differences in demographics and other characteristics among participants in a phone survey versus non-participants, and among participants in a phone survey versus those in a face-to-face survey, within a rural population with a high HIV burden. Specifically, the study compares individuals with and without access to mobile phones, as well as participation rates between previous face-to-face interviews and the current mobile phone survey.

## Methods

### Study setting

The mobile phone study was conducted within the Rakai Community Cohort Study (RCCS), an ongoing population-based, prospective, open cohort that has enrolled permanent and transient residents aged 15–49 years in approximately 40 communities in south-central Uganda. Included communities are predominantly agrarian; the cohort also includes peri-urban townships dominated by the informal trade sector and fishing communities along Lake Victoria. The study location includes the districts of Rakai, Kyotera, Masaka and Lyantonde, with a Tanzanian border post at Mutukula. All the included districts have major stopovers for cross-border truck drivers. At the time of this study, Uganda was experiencing its first wave of COVID-19 infections, resulting largely from cross-border transmissions.

Each RCCS round, conducted approximately every 2 years, includes a census in each community to enumerate household members’ demographics and household possessions, including mobile phone ownership and access. The most recent RCCS rounds that are part of this analysis occurred during October 2016–May 2018 (Round 18) and June 2018–August 2020 (R19 with COVID-19 disruptions). Mobile phone numbers have been recorded since 2011. [21] Three to 4 weeks after the census, all-consenting adults and assenting minors respond to a behavioral survey and are offered an HIV rapid test including pre- and post-test HIV counseling. Over 95% of participants accept an HIV test. [20] All participants who test positive for HIV but are not enrolled in HIV care are immediately initiated on antiretroviral therapy (ART) via the United States President’s Emergency Plan for AIDS Relief (PEPFAR).

### Participants and sampling

Eligibility for face-to-face surveys required individuals listed in the recent RCCS census, residing within RCCS boundaries, aged 15–49, and living in the community for at least 6 months (or 2 months with intent to stay). Survey participants were those consenting to be interviewed by the RCCS team. The RCCS participants were eligible for the present rapid phone survey if they were 18 years or older, had participated in the RCCS survey at least once since 2017; provided a mobile phone number for self or a close contact, baseline demographics, and behavioral data at their last RCCS survey, and had their HIV status determined at their last RCCS survey. All eligible people living with HIV who were RCCS participants were sampled and then matched one-to-one by age (±5 years) and community with an HIV-negative control participant. Most phone survey participants were from communities that had recently completed a face-to-face RCCS survey visit within Round 19. In communities that had not yet been surveyed in Round 19 due to the lockdown, sampling was based on previously obtained RCCS data from Round 18 (Supplementary table S1a). Respondents’ HIV status was not disclosed or discussed during contact for phone interviews. Interviewers were provided with a sample list that masked participants’ HIV status. As a result, interviewers attempted to contact each participant sequentially without prior knowledge of their HIV status. This approach likely led to varying enrollment numbers across different HIV serostatus groups, age, and communities.

### Training, recruitment, and data collection

Experienced interviewers (from the RCCS) conducted the phone survey. Prior to the survey, 39 interviewers and 5 data quality control officers underwent a three-day training on the protocol, survey tools, telephone interviewing techniques, and audio recording of informed consent [22]. Role-plays and pre-test interviews were used to assess audibility, manage time, participant identification techniques, and strengthening of probing skills. Interviewers were trained to counsel and refer participants with COVID-19 symptoms. Interviews were conducted by interviewers who were of the same sex as the participant.

The interviewers attempted to contact participants using the respondent’s most recent reported phone number or immediate contact. When participants had more than one reported phone number, interviewers attempted calling all the numbers. When the call was answered, the interviewer introduced themselves and asked if the participant was willing to respond to a short survey. If potential participants responded affirmatively, the interviewer confirmed their identity using a checklist based on RCCS records. Participants who were unable to speak at the time of the call were offered an appointment to be recontacted at their convenience. Interviewers administered a verbal, audio-recorded informed consent prior to the interview. Participants could decline enrollment or stop the interview at any time. Up to three attempts were made to contact participants in instances of unanswered phone calls, phones being off, dropped calls, or inaudibility.

Participants were asked questions about access to essential medical care and the effects of COVID-19 and related control measures. Most questions were open-ended and unprompted, allowing participants to answer in whatever way made sense to them. Questions had pre-coded answers, allowing interviewers to select the answers that best captured the participant’s response. Data were electronically captured using a Microsoft Access database. Each completed survey was checked by a quality control officer to ensure completeness and consistency.

### Primary outcomes

Primary factors assessed included phone access and participation in face-to-face and phone interviews. Other factors included sex, age, community type, education level, employment type, marital status, and HIV status. Phone access was defined as owning a mobile phone number or providing contact through a household member.

### Statistical analysis

The study conducted descriptive analyses focusing on demographic variables and key outcomes to evaluate phone access and participation rates across different survey modalities. Specifically, it compared participants who completed the phone survey with those who were unreachable after three attempts. The study used logistic regression models to estimate odds ratios (OR) and 95% confidence intervals (CI) for factors influencing survey participation, which was a binary outcome (1 for participation, 0 for non-participation). The key independent variable was the interview type (face-to-face or phone). Additional covariates included sex, age group, community type, education level, occupation, marital status, and HIV status. Robust standard errors were applied to account for clustering within the data at the individual level. To identify factors associated with phone access in both face-to-face and phone interviews, modified Poisson regression models were utilized. We adjusted for several key covariates, including age, sex, socioeconomic status, and education level, as these variables were identified as potential confounders in the relationship between the predictors and our outcomes of interest. These models were used to estimate unadjusted and adjusted prevalence ratios (aPR), along with their 95% confidence intervals (CI). All the statistical analyses were performed using Stata, version 15 (Stata Corp LLC, College Station, TX).

### Definitions

#### RCCS face-to-face survey participant

Persons who were contacted by the RCCS survey team and consented to be interviewed (enrolled) in the RCCS.

#### Phone survey eligible

PLWH within the RCCS, aged 18–49, who reported ownership of a phone in the most recent RCCS census, and have participated in Round 18 or 19 RCCS face-to-face survey and sampled HIV-negative controls matched on community.

#### Phone survey participation

Phone survey eligible persons who were successfully contacted and interviewed during the present phone survey.

#### Phone access

Persons who owned a mobile phone number or provided a contact number through a member within the same household.

#### RCCS round

These refer to RCCS study visits and face-to-face surveys, which occur approximately every 2 years within the cohort study communities. At the time of the present phone survey, RCCS Round 19 data collection was ongoing but disrupted due to COVID-19. Part of the main analysis compares data from the phone survey with data from RCCS Round 18, which was the most recently completed study visit prior to the COVID-19 pandemic.

## Results

**Figure 1.**
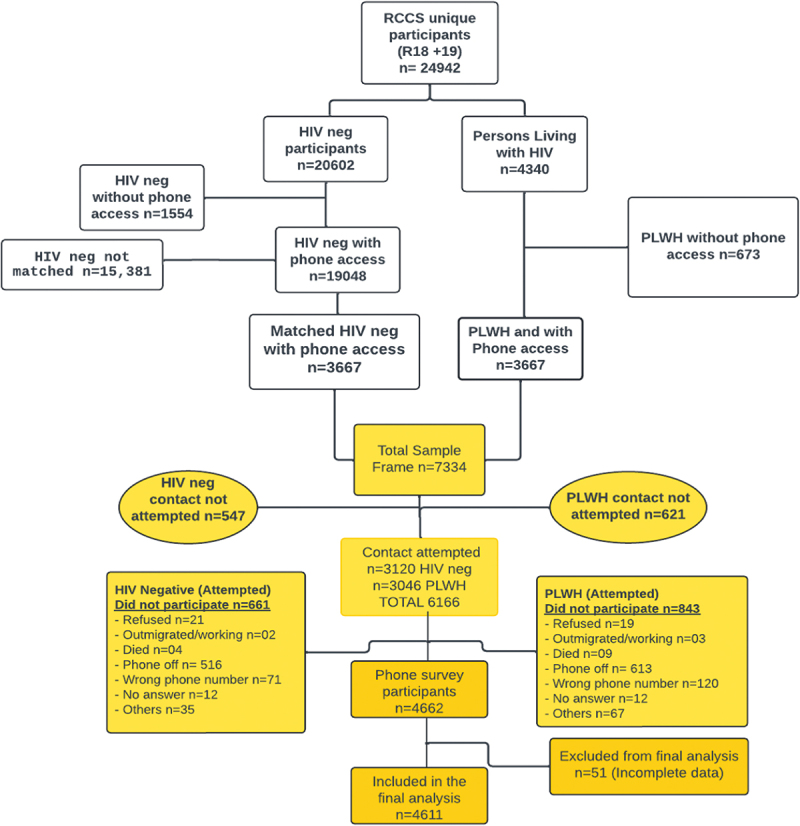
Illustration of the sampling for the phone survey in the context of RCCS. Overall 24,942 unique individuals participated in the RCCS for rounds 18 and 19. A total of *n* = 3,667 PLWH had access to phones and were matched 1:1 to an HIV-negative control (*n* = 7,334 sample frame). For a total of 547 HIV negatives and 621 PLWH contact was not attempted for logistical reasons related to changes in COVID-19 restrictions. Therefore, *n* = 6,166 participants were attempted for inclusion in the phone survey (PLWH *n* = 3,120, and HIV negative matches *n* = 3,046). Among these, *n* = 4,662 were successfully contacted and *n* = 4,611 were included in the final analysis.

Phone survey data were collected between 25 May 2020, and 7 August 2020, using phone interviews. Out of a total of 24,942 unique individuals who participated in either Round 18 or Round 19; 4,340 participants were living with HIV at a previous face-to-face survey round. Out of these, 3,667 had phone access (84.5%) and were then matched by age and community to HIV-negative participants, resulting in a sample frame of 7,334 participants. Matching was performed during sampling, and interviewers, who were blinded to participants’ HIV status, conducted the interviews without considering sero-status. Due to logistical reasons (COVID restrictions being lifted necessitating the RCCS survey to resume) contact was attempted with only 3,046 PLHW and 3,120 hIV negative participants resulting in 6,166 potential phone survey participants. Out of these, 1,504 people (24.4%) did not enroll in the study. Most of those who did not enroll (*n* = 1,129, 75.1%) had their phones off at the third and final attempt to contact them. Only 2.7% (*n* = 40) refused participation. A total of 4,662 (75.7%) participants were successfully contacted. However, in the final analysis on social and health outcomes, 51 (1.1%) of the contacted participants were excluded due to incomplete data, leaving a total of 4,611 in the analysis (Figure 1).

Among those we failed to reach (n = 1,504), 843 (56.2%) were PLWH. We compared the reasons for non-participation by HIV serostatus and found no significant differences (data not shown). In comparison to those who participated in the phone survey and those who were unreachable, men were more likely than women not to be reached (adjusted OR 1.42. 95% CI 1.23–1.63,  < 0.001), and so were persons resident in agrarian communities relative to those in trading peri-urban communities (adjusted OR 1.29, 95% CI 1.09–1.52, *p* = 0.002) and persons with secondary education or above were also more likely not to be reached (adjusted OR 1.56, 95% CI, 1.35–1.80, *p* < 0.001).

### Mobile phone access

For the analysis of phone access, we used only Round 19 data (*n* = 17,281). Overall, 15,594 (90.2%) had access to mobile phones. Among RCCS participants, a significantly larger proportion of women (92.2%) had access to mobile phones compared to men (88.0%), *p* < 0.001. Fishing village residents had lower access to mobile phones compared to residents in agrarian and trading communities (79.2% vs 93.7% and 93.6%, respectively) (adjusted PR = 0.88, 95% CI: 0.87–0.90, *p* < 0.001) (Table 1). In the adjusted model, women still had a slightly higher prevalence of phone access compared to men (adjusted PR = 1.04, 95% CI: 1.03–1.04, *p* < 0.001). Higher education level was associated with higher phone access. Being a person living with HIV was associated with a lower prevalence of phone access (adjusted PR = 0.96, 95% CI: 0.94–0.98, *p* < 0.001) (Table 2).

**Table 1.** Phone access in Round 19 of the Rakai Community Cohort Study by demographic characteristics.

	Phone access			
	Yes	No	Total	*P* value
**Overall**	**15,594 (90.2)**	**16,87 (9.8)**	**17,281**	
**Sex**				
Men	7,093 (88)	963 (12)	8,056	< 0.001
Women	8,501 (92.2)	724 (7.8)	9,225	
**Age group**				
15–17	1,650 (92.7)	129 (7.3)	1,779	< 0.001
18–24	3,765 (92.9)	286 (7.1)	4,051	
25–34	4,778 (89.5)	558 (10.5)	5,336	
35–44	4,114 (88.7)	524 (11.3)	4,638	
45+	1,287 (87.1)	190 (12.9)	1,477	
**Community type**				
Trading	4,448 (93.6)	303 (6.4)	4,751	< 0.001
Fishing	3,222 (79.2)	847 (20.8)	4,069	
Agrarian	7,924 (93.7)	537 (6.3)	8,461	
**Education level**				
Primary and below	9,470 (86.7)	1,459 (13.3)	10,929	< 0.001
Secondary and above	6,124 (96.4)	228 (3.6)	6,352	
**Occupation**				
Agriculture/housewife	6,109 (90.5)	644 (9.5)	6,753	< 0.001
Bar/restaurant	601 (88.3)	80 (11.7)	681	
Truck/Boda-boda	386 (96)	16 (4)	402	
Trade/shop	2,419 (94.2)	149 (5.8)	2,568	
Others	6,079 (88.4)	798 (11.6)	6,877	
**Marital status**				
Married	8,944 (92.5)	720 (7.5)	9,664	< 0.001
Previously married	2,347 (78.7)	634 (21.3)	2981	
Never married	4,300 (92.8)	333 (7.2)	4,633	
**HIV status**				
Negative	13,024 (92)	1,137 (8)	14,161	< 0.001
Positive	2,570 (82.4)	550 (17.6)	3,120

**Table 2.** A comparison of participation rates by interview model and a logistic model for participation in the interview.

			Unadjusted		Adjusted*	
	Face to face interview	Phone interview	Odds ratio (95% CI)	*P* value	Odds ratio (95% CI)	*P* value
**Interview Type**			1		1	
Face to face interview			1		1	
Phone interview			0.66 (0.09–4.67)	0.675	0.56 (0.08–3.90)	0.554
**Sex**						
Men	6,150/7,350 (83.7)	2,687/3,509 (76.6)	1		1	
Women	5,109/6,399 (79.8)	1,924/2,653 (72.5)	1.25 (1.13–1.39)	<0.001	1.18 (1.11–1.26)	< 0.001
**Age group**						
18–24	2,209/3,120 (70.8)	141/241 (58.5)	1		1	
25–34	3,946/4,740 (83.2)	1,390/1,998 (69.6)	1.64 (0.85–3.17)	0.143	1.63 (1.48–1.80)	< 0.001
35–44	3,837/4,425 (86.7)	2,038/2,658 (76.7)	2.09 (0.93–4.73)	0.076	2.13 (1.82–2.49)	< 0.001
45+	1,267/1,464 (86.5)	1,042/1,265 (82.4)	2.37 (1.01–5.54)	0.048	2.43 (1.75–3.37)	< 0.001
**Community type**						
Trading	3,611/4,465 (80.9)	1,338/1,724 (77.6)	1		1	
Fishing	2,460/3,037 (81)	1,530/2,300 (66.5)	0.85 (0.83–0.86)	<0.001	0.87 (0.86–0.89)	< 0.001
Agrarian	5,188/6,247 (83)	1,743/2,138 (81.5)	1.00 (0.99–1.01)	0.944	1.01 (1.00–1.02)	0.079
**Education level**						
Primary and below	7,576/9,010 (84.1)	3,063/4,250 (72.1)	1		1	
Secondary and above	3,683/4,739 (77.7)	1,548/1,912 (81)	0.91 (0.62–1.34)	0.625	0.97 (0.77–1.22)	0.777
**Occupation**						
Agriculture/housewife	4,211/4,817 (87.4)	1,795/2,288 (78.5)	1		1	
Bar/restaurant	393/478(82.2)	298/437 (68.2)	0.56 (0.39–0.81)	0.002	0.75 (0.62–0.89)	0.001
Truck/Boda-boda	109/138 (79)	109/139 (78.4)	0.68 (0.52–0.88)	0.003	0.92 (0.65–1.28)	0.606
Trade/shop	1,666/2,030 (82.1)	991/1,286 (77.1)	0.74 (0.68–0.80)	<0.001	0.86 (0.69–1.06)	0.162
Others	4,880/6,286 (77.6)	1,418/2,012 (70.5)	0.58 (0.43–0.78)	<0.001	0.77 (0.70–0.84)	< 0.001
**Marital status**						
Married	7,094/8,331 (85.2)	3,183/41,36 (77)	1		1	
Previously married	1,898/2,195 (86.5)	1,092/1,549 (70.5)	0.85 (0.60–1.19)	0.336	0.89 (0.74–1.09)	0.265
Never married	2,267/3,223 (70.3)	336/477 (70.4)	0.51 (0.28–0.92)	0.027	0.67 (0.61–0.74)	< 0.001
**HIV status**						
Negative	9,094/11,171 (81.4)	2,429/3,116 (78)	1		1	
No result	17/22 (77.3)					
Positive	2,148/2,556 (84)	2,182/3,046 (71.6)	0.82 (0.35–1.90)	0.639	0.83 (0.76–0.91)	< 0.001

*In the model, adjustments were made for sex, age, community type, occupation, marital status, and HIV serostatus.

### Participation rates across the survey modality

In comparing survey participation rates between the face-to-face and phone surveys, we discovered that participation was slightly higher in the face-to-face survey overall (81.9%) than in the phone survey (74.8%). In the model, this was not statistically significant (adjusted OR = 0.56, 95% CI, 0.08–3.90 *p* = 0.55).

Women had a higher participation rate than men in both the face-to-face survey (83.7% and 79.8%) and phone interviews (76.6% vs 72.5%) (adjusted OR 1.18, 95% CI, 1.11–1.29, *p* < 0.001). Among the younger participants aged 18–24 years old, the participation rate was higher in the face-to-face survey compared to the phone survey (70.8% vs 58.5%). Similarly, for participants aged 25–34 years, the face-to-face survey also showed a higher participation rate compared to the phone survey (83.2% vs 69.6%). We observed that as age increases, participation rate also increases across both survey models. In the logistic model, relative to those aged 18–24, among those 25–34 (adjusted OR 1.63, 95% CI, 1.48–1.80 p < 0.001), among those 35–44 (adjusted OR 2.13, 95% CI, 1.82–2.49 p < 0.001), among those 45+ (adjusted OR 2.43, 95% CI, 1.75–3.37, p < 0.001).

In HIV high-risk fishing communities, the participation rate was notably higher in face-to-face surveys compared to phone surveys (81.0% vs 66.5%). Relative to participants in trading communities, the fisherfolk population is less likely to participate in surveys (adjusted OR 0.87, 95% CI; 0.86–0.89 *p* < 0.001). This difference in survey participation is not observed among the agrarian population (adjusted OR 1.01, 95% CI,1.00–1.02, *p* < 0.001)

When considering education levels, participants with primary education and below were more likely to participate in face-to-face surveys than in phone surveys (84.1% vs 72.1%). Conversely, among those with secondary school education and above, participation was slightly higher in phone surveys compared to face-to-face surveys (80.9% vs 77.7%). When participation was modeled, we did not observe significant difference in survey participation based on education level (adjusted OR 0.97, 95% CI, 0.77–1.22, *p* = 078).

Regarding occupation, participation rate was higher in the face-to-face surveys than in the phone surveys among ba/restaurant workers (82.4% vs 68.2%). Relative to participants in subsistence agriculture and housewives, bar workers were less likely to participate in surveys (adjusted OR 0.75, 95% CI, 0.62–0.89, *p* < 0.001). Participation in surveys among persons in other occupations was comparable.

Previously married individuals’ participation rate was higher in the face-to-face than in the phone survey (86.5% vs 70.5%). Relative to married individuals, participants who have never married were less likely to participate in surveys (adjusted OR 0.67, 95% CI, 0.61–0.74, *p* < 0.001).

Among persons living with HIV, the participation rate was higher in the face-to-face mode than in the phone survey (84.0% vs 71.6%). Relative to HIV negative persons, PLWH were less likely to participate in surveys (adjusted OR 0.83, 95% CI, 0.76–0.91, *p* < 0.001).

## Discussion

This study highlights the practicability of rapidly collecting data during an emergency through mobile phone surveys in rural Uganda, achieving high participation and coverage despite some demographic differences among participants. Even when calling from an unknown number, it was possible to conduct a 30-minute conversation to obtain informed consent and interview respondents about health and social issues. This is notable given the survey community's limited mobile phone network coverage, limited electricity access (for battery charging), and prevalent fear of scammers [23,24].

This study revealed that 90.2% of residents in this rural setting have access to mobile phones. We observed gender disparities in accessibility, where women had slightly higher access (92.2%) relative to men (88.0%). Another recent study in Uganda that enrolled only women to understand the feasibility of using mobile phones to support Human Papilloma Virus self-care (HPV-SC) corroborates high mobile phone ownership and access rates in rural eastern Uganda, with combined ownership and access exceeding 90% in some areas. [25] However, attention is needed for individuals without access, who appear distinct from those with access. Historically, women have had limited access to mobile phones relative to men, and most of those studies have restricted their analysis on ownership rather than on broader access [21,26].

There are varying definitions of access in the context of mobile phone studies. In our study, access was based on a mobile phone present in the household. Measuring access based on the presence of a mobile phone in a household can indeed lead to an overestimation of utilizable access. This method assumes that the phone is equally accessible to all household members, which may not be the case. In some households, especially in contexts where gender or social inequalities are prevalent, the primary control of the mobile phone might rest with certain members, typically men or individuals of higher status. Women, children, or marginalized groups within those households may have limited or no access to the phone, and even if they do, their usage might be restricted by social norms. Conversely, the absence of a mobile phone in the household might not completely capture a lack of access. Individuals might still have occasional or shared access through neighbors, community phones, or other means. This could lead to an underestimation of access rates. Given these complexities, it is essential to establish contextually appropriate definitions and methods for measuring access.

The high access and participation rates may be attributed to the common use of mobile phones for informal trade and mobile banking in the region [27]. We obtained a 74.8% participation rate in our phone survey, which is comparable to other phone surveys conducted during the Ebola epidemic in Sierra Leone and Liberia [28,29]. The use of a pre-existing sampling frame and prior contact likely contributed to higher response rates in this study compared to methods like random digit dialing or using lists from service providers [30]. This finding indicates that existing population surveillance cohorts, such as the RCCS, are suitable for conducting mobile phone surveys during public health emergencies.

Approximately 75% of the sample population we attempted to contact for the phone survey had their phones turned off. Electrification rates in rural Uganda (around 18%) are substantially lower than in urbanized areas (around 60%) which affects people’s ability to charge their devices [31–33] and can lead to lapses in network coverage [34]. In this study, participation rates were lowest in fishing communities, possibly due to disproportionately lower mobile phone network coverage in these areas [35]. In addition, one major fishing community included in this study was a hotspot for cross-border COVID-19 transmissions leading residents to switch off their phones to avoid contact tracing [36,37]. Despite generally lower phone access among women compared to men [38,39], participation in our survey was higher for women than men. These dynamics are crucial for interpreting survey results accurately and ensuring that efforts to engage all demographic groups effectively address potential barriers to participation. It also underscores the importance of tailoring survey methodologies to diverse participant needs and preferences.

It has been widely reported that women show relatively higher participation rates across various survey modes. A study in Nigeria explored women’s high acceptability to mobile phone interview. The study reported that women found telephone interview procedures easy and perceived that the call offered privacy. In addition, women felt more able to re-arrange interview times on the phone compared to a face-to-face interview, they valued the increased autonomy as they were often busy with household chores and could rearrange to a more convenient time [23,40].

A comparison of participation rates for the phone survey vs. the RCCS face-to-face revealed that participation rates were slightly higher in the face-to-face survey than in the phone survey. Although in this study, the difference was not significant, we still considered it an important observation. This finding is consistent with a previous study that reported a lower response rate in phone surveys relative to face-to-face surveys has been reported in sub-Saharan Africa before [41]. This lower response rate is majorly attributed to nonresponse due to non or poor contact due to network coverage and quality, battery charge, and whether the respondent is willing to answer an unknown caller or even provide information about themselves at the risk of responding to a scammer.

Phone survey sampling may not be representative of the general population, a challenge reported elsewhere. [40] Strategies to address the limited generalizability of phone sampling in the future work include employing effective sampling methods and statistical techniques to adjust for selection bias. In our phone survey, we saw that it was more difficult to reach participants who were younger, women, living with HIV and residing in fishing communities, compared to face-to-face surveys where it was more difficult to reach those with higher education.

This study is not without limitations. The study compares participation rates across two different survey data collection modes; however, the tools used for each mode were different. The RCCS administers a detailed face-to-face personal behavioral survey that lasts for about 60 minutes, while the phone survey tool was brief (lasting about 30 minutes) and focused on broader questions about COVID-19. This brevity could have made the phone survey more attractive to respondents, resulting in a higher participation rate in the phone survey than would have occurred with the original 60-minute tool. This is not unique to this study; in general, phone surveys tend to be relatively shorter than self-administered and face-to-face surveys. The timing of the two surveys was also different, as the phone survey happened at a time when most respondents were at home during the COVID-19 lockdown. This could have resulted in higher participation rates in the phone survey than would ordinarily occur. In addition, the two survey approaches used different sampling criteria; the phone survey used a matched case–control sampling approach based on HIV status that was more restricted than the RCCS, which enrolls all eligible consenting residents. Finally, due to factors outside our control, with COVID-19 restrictions eased slightly before the end of the phone survey, we had to stop data collection before we could attempt all the sampled participants.

## Conclusion

We find that using mobile phone surveys is possible during public health emergencies. Participation rates were somewhat lower compared to face-to-face interviews, especially among people living with HIV. However, generalizability remains a challenge as it is limited to ongoing population-based surveillance programs. It is essential to understand how using a phone survey might affect participation rates in different groups.

## Supplementary Material

Tables Phone survey_25_Sept 2024__revised3_clean.docx

## Abbreviations

ART: Antiretroviral therapy
PLWH: People living with HIV
RCCS: Rakai Community Cohort Study
