# Correction

**DOI:** 10.1080/16549716.2025.2531701

**Published:** 2025-07-22

**Article title:** Use of mobile phones to collect data on COVID-19: phone access and participation rates, in Rakai, Uganda

**Authors:** Robert Ssekubugu, Anthony Ndyanabo, Fredrick Makumbi, Anna Mia Ekström, Laura Beres, Grace Nalwoga Kigozi, Hadijja Nakawooya, Joseph Ssekasanvu, Maria J Wawer, Fred Nalugoda, Nelson Sewankambo, Victor Ssempijja, Betty Nantume, David Serwadda, Godfrey Kigozi, Ronald H. Gray, Larry W. Chang, M. Kate Grabowski, Helena Nordenstedt and Joseph Kagaayi

**Journal:** *Global Health Action*

**Bibliometrics:** Volume 17, Number 1, e2419160

**DOI:** http://dx.doi.org/10.1080/16549716.2024.2419160

When this article was originally published, the face-to-face interview and phone interview comparison data for men and women in Table 2 were reversed.

This has now been corrected.

