# Supplementary material for: Use of mobile phones to collect data on COVID-19: phone access and participation rates, in Rakai, Uganda
Source: Glob Health Action. 2024 Nov 12;17(1):2419160. doi: 10.1080/16549716.2024.2419160 (PMC11559023; doi:10.1080/16549716.2024.2419160)
Supplement: Tables Phone survey_25_Sept 2024__revised3_clean.docx [file ZGHA_A_2419160_SM0313.docx]

*Table 1: Phone access in Round 19 of the Rakai Community Cohort Study by demographic characteristics.*

|  | **Phone access** | | | |
| --- | --- | --- | --- | --- |
|  | **Yes** | **No** | **Total** | **P value** |
| **Overall** | **15594(90.2)** | **1687(9.8)** | **17281** |  |
| **Sex** |  |  |  |  |
| Men | 7093(88) | 963(12) | 8056 | <0.001 |
| Women | 8501(92.2) | 724(7.8) | 9225 |  |
| **Age group** |  |  |  |  |
| 15-17 | 1650(92.7) | 129(7.3) | 1779 | <0.001 |
| 18-24 | 3765(92.9) | 286(7.1) | 4051 |  |
| 25-34 | 4778(89.5) | 558(10.5) | 5336 |  |
| 35-44 | 4114(88.7) | 524(11.3) | 4638 |  |
| 45+ | 1287(87.1) | 190(12.9) | 1477 |  |
| **Community type** |  |  |  |  |
| Trading | 4448(93.6) | 303(6.4) | 4751 | <0.001 |
| Fishing | 3222(79.2) | 847(20.8) | 4069 |  |
| Agrarian | 7924(93.7) | 537(6.3) | 8461 |  |
| **Education level** |  |  |  |  |
| Primary and below | 9470(86.7) | 1459(13.3) | 10929 | <0.001 |
| Secondary and above | 6124(96.4) | 228(3.6) | 6352 |  |
| **Occupation** |  |  |  |  |
| Agriculture/housewife | 6109(90.5) | 644(9.5) | 6753 | <0.001 |
| Bar/restaurant | 601(88.3) | 80(11.7) | 681 |  |
| Truck /Boda-boda | 386(96) | 16(4) | 402 |  |
| Trade/shop | 2419(94.2) | 149(5.8) | 2568 |  |
| Others | 6079(88.4) | 798(11.6) | 6877 |  |
| **Marital status** |  |  |  |  |
| Married | 8944(92.5) | 720(7.5) | 9664 | <0.001 |
| Previously married | 2347(78.7) | 634(21.3) | 2981 |  |
| Never married | 4300(92.8) | 333(7.2) | 4633 |  |
| **HIV status** |  |  |  |  |
| Negative | 13024(92) | 1137(8) | 14161 | <0.001 |
| Positive | 2570(82.4) | 550(17.6) | 3120 |  |

Table 2: Comparing of participation rates by interview model and a logistic model for participation in the interview

|  | **Face to face interview** | **Phone interview** |  | **Unadjusted** | | **Adjusted*** | |
| --- | --- | --- | --- | --- | --- | --- | --- |
| **Interview Type** |  |  |  | **Odds ratio**  **(95% CI)** | **P value** | **Odds ratio**  **(95% CI)** | **P value** |
| Face to face interview |  |  |  | 1 |  | 1 |  |
| Phone interview |  |  |  | 0.66(0.09-4.67) | 0.675 | 0.56(0.08-3.90) | 0.554 |
| **Sex** |  |  |  |  |  |  |  |
| Men | 6150/7350(83.7) | 2687/3509(76.6) |  | 1 |  | 1 |  |
| Women | 5109/6399(79.8) | 1924/2653(72.5) |  | 1.25(1.13-1.39) | <0.001 | 1.18(1.11-1.26) | <0.001 |
| **Age group** |  | |  |  |  |  |  |
| 18-24 | 2209/3120(70.8) | 141/241(58.5) |  | 1 |  | 1 |  |
| 25-34 | 3946/4740(83.2) | 1390/1998(69.6) |  | 1.64(0.85-3.17) | 0.143 | 1.63(1.48-1.80) | <0.001 |
| 35-44 | 3837/4425(86.7) | 2038/2658(76.7) |  | 2.09(0.93-4.73) | 0.076 | 2.13(1.82-2.49) | <0.001 |
| 45+ | 1267/1464(86.5) | 1042/1265(82.4) |  | 2.37(1.01-5.54) | 0.048 | 2.43(1.75-3.37) | <0.001 |
| **Community type** |  |  |  |  |  |  |  |
| Trading | 3611/4465(80.9) | 1338/1724(77.6) |  | 1 |  | 1 |  |
| Fishing | 2460/3037(81) | 1530/2300(66.5) |  | 0.85(0.83-0.86) | <0.001 | 0.87(0.86-0.89) | <0.001 |
| Agrarian | 5188/6247(83) | 1743/2138(81.5) |  | 1.00(0.99-1.01) | 0.944 | 1.01(1.00-1.02) | 0.079 |
| **Education level** |  |  |  |  |  |  |  |
| Primary and below | 7576/9010(84.1) | 3063/4250(72.1) |  | 1 |  | 1 |  |
| Secondary and above | 3683/4739(77.7) | 1548/1912(81) |  | 0.91(0.62-1.34) | 0.625 | 0.97(0.77-1.22) | 0.777 |
| **Occupation** |  |  |  |  |  |  |  |
| Agriculture/housewife | 4211/4817(87.4) | 1795/2288(78.5) |  | 1 |  | 1 |  |
| Bar/restaurant | 393/478(82.2) | 298/437(68.2) |  | 0.56(0.39-0.81) | 0.002 | 0.75(0.62-0.89) | 0.001 |
| Truck /Boda-boda | 109/138(79) | 109/139(78.4) |  | 0.68(0.52-0.88) | 0.003 | 0.92(0.65-1.28) | 0.606 |
| Trade/shop | 1666/2030(82.1) | 991/1286(77.1) |  | 0.74(0.68-0.80) | <0.001 | 0.86(0.69-1.06) | 0.162 |
| Others | 4880/6286(77.6) | 1418/2012(70.5) |  | 0.58(0.43-0.78) | <0.001 | 0.77(0.70-0.84) | <0.001 |
| **Marital status** |  |  |  |  |  |  |  |
| Married | 7094/8331(85.2) | 3183/4136(77) |  | 1 |  | 1 |  |
| Previously married | 1898/2195(86.5) | 1092/1549(70.5) |  | 0.85(0.60-1.19) | 0.336 | 0.89(0.74-1.09) | 0.265 |
| Never married | 2267/3223(70.3) | 336/477(70.4) |  | 0.51(0.28-0.92) | 0.027 | 0.67(0.61-0.74) | <0.001 |
| **HIV status** |  |  |  |  |  |  |  |
| Negative | 9094/11171(81.4) | 2429/3116(78) |  | 1 |  | 1 |  |
| No result | 17/22(77.3) |  |  |  |  |  |  |
| Positive | 2148/2556(84) | 2182/3046(71.6) |  | 0.82(0.35-1.90) | 0.639 | 0.83(0.76-0.91) | <0.001 |

**In the model, adjustments were made for sex, age community type, occupation, marital status and HIV serostatus*
